# Androgen receptor alpha deficiency impacts aromatase expression in the female cichlid brain

**DOI:** 10.1098/rsos.240608

**Published:** 2024-07-03

**Authors:** Mariana S. Lopez, Beau A. Alward

**Affiliations:** ^1^ Department of Psychology, University of Houston, Houston, TX 77204, USA; ^2^ Department of Biology and Biochemistry, University of Houston, Houston, TX 77204, USA

**Keywords:** androgen receptor, aromatase, neuroendocrinology, teleost

## Abstract

Steroid hormones bind to specific receptors that act as transcription factors to modify gene expression in the brain to regulate physiological and behavioural processes. The specific genes controlled by steroid hormones in the brain are not fully known. Identifying these genes is integral to establishing a comprehensive understanding of how hormones impact physiology and behaviour. A popular organism for answering this question is the cichlid fish *Astatotilapia burtoni*. Recently, CRISPR/Cas9 was used to engineer *A. burtoni* that lack functional androgen receptor (AR) genes encoding ARα. ARα mutant male *A. burtoni* produced fewer aggressive displays and possessed reduced expression of the gene encoding brain-specific aromatase, *cyp19a1*, in the ventromedial hypothalamus (VMH), an aggression locus. As a follow-up, we investigated whether ARα deficiency affected *cyp19a1* expression in female *A. burtoni* using the same genetic line. We find that female *A. burtoni* possessing one or two non-functional ARα alleles had much higher expression of *cyp19a1* in the preoptic area (POA), while females with one non-functional ARα allele possessed lower expression of *cyp19a1* in the putative fish homologue of the bed nucleus of the stria terminalis (BNST). Thus, ARα may have a sex-specific role in modifying *cyp19a1* expression in the teleost POA and BNST, regions that underlie sex differences across vertebrates.

## 1. Introduction

Steroid hormones regulate physiological and behavioural traits across vertebrate species [1,2]. In the brain, steroid hormones act on specific receptors to alter gene expression that leads to changes in certain traits [2–4]. The actions of sex steroid hormones such as androgens and oestrogens have been shown to underlie sex differences in brain gene expression, as well as neuroplasticity and behaviour across vertebrate species. Sex steroid hormones include androgens like testosterone, dihydrotestosterone (DHT) and 11-ketotestosterone (11-KT), which act on androgen receptors (ARs); as well as oestrogens such as estradiol that act on oestrogen receptors (ERs). Also present throughout the brain is the enzyme aromatase—encoded by the gene *cyp19a1*—which converts testosterone to estradiol.

Gaps in our knowledge of the role of steroid hormones remain, however. For example, while it is known that sex steroid hormones modulate brain and behaviour, the specific genes in the brain that are involved in these processes have not been fully characterized. Thus, the use of novel steroid receptor mutant animals may assist in showing clear links between steroid hormone action and behaviour.

The African cichlid *Astatotilapia burtoni* is one such organism in which the generation of novel mutants may aid in understanding the links between steroid hormone action and behaviour. Due to a whole genome duplication, *A. burtoni* have two copies of various steroid hormone-related genes including ARs, ERs and aromatase [5,6]. In *A. burtoni,* like other teleosts, brain-specific aromatase (*cyp19a1*) is expressed primarily in brain and pituitary, while gonadal aromatase (*cyp19a1a*) is expressed primarily in the gonads and interrenal glands [5–7]. The specific functions of gonadal aromatase and brain aromatase, however, are poorly understood. In addition, recent studies in this and other teleost species show a strong connection between androgens, ARs, *cyp19a1* expression and social behaviours in males [8–10]. We recently generated novel AR mutants in *A. burtoni* and showed that ARα mutant males produced fewer aggressive displays compared with wild-type (WT) males [11] and possessed reduced *cyp19a1* expression in the putative fish homologue of the ventromedial hypothalamus (VMH) [12], a locus known to control aggression [13]. It was also found that ARα co-expressed with *cyp19a1* throughout cells in the brain in both males and females [12], suggesting ARα may affect *cyp19a1* expression in both sexes.

Here, we investigated whether ARα deficiency in female *A. burtoni* may impact *cyp19a1* expression in a similar way as in ARα-deficient males. Similar to our previous study [12], we analysed *cyp19a1* expression in the social behaviour network (SBN), a highly conserved circuit that integrates environmental cues and modifies physiological and behavioural processes across vertebrate species [13–16]. We quantified *cyp19a1* expression differences among female ARα mutant and WT *A. burtoni* in the following SBN brain regions: the preoptic area (POA), subcomissural nucleus of the ventral telencephalon (Vs), ventral nucleus of the ventral telencephalon (Vv), lateral tuberal nucleus (VTn/NLT) and anterior tuberal nucleus (ATn); the putative mammalian homologues are the bed nucleus of the stria terminalis (BNST) and medial amygdala (meAMY); lateral septum (LS); anterior hypothalamus (AH); and the VMH, respectively. We find that ARα-deficient *A. burtoni* females show differences compared with WT females in *cyp19a1* expression in two different SBN nuclei—the POA and Vs—two regions that regulate sexually dimoprhic social behaviours.

## 2. Methods

### 2.1. Animal subjects and housing

Laboratory-bred fish used for these experiments originated from a stock from Lake Tanganyika, Africa collected in the 1980s that were subsequently bred over multiple generations and have been kept in laboratory conditions simulating their natural habitat (25°C; 12 h day : 12 h night cycle) [17,18] for hundreds of generations from the original stock in accordance with Association for Assessment and Accreditation of Laboratory Animal Care standards. All experimental procedures were approved by the University of Houston Institutional Animal Care and Use Committee (Protocol no. 202000001).

#### 2.1.1. ARα mutant females

We used female *A. burtoni* with a mutant ARα (accession no. NW_005179415) allele. The process used for generating these mutants is previously described in full detail by Alward *et al.* [11]. Briefly, Cas9 was directed towards two regions within exon 1 using two single-guide RNAs (sgRNAs) targeting sequence ARα gene sequence. G1 offspring from injected fish carried a frameshift deletion within exon 1 of ARα of 50 bp. G1 fish were then outcrossed to unrelated WT fish and then intercrossed with heterozygous mutants to obtain biallelic ARα mutants (ARα^d50/d50^, hereon referred to as ARα^KO^), Het ARα mutants (ARα^d50/+^, hereon referred to as ARα^Het^) and ARα WT (ARα^+/+^, hereon referred to as ARα^WT^). The stable line from which ARα experimental fish are used is the result of several separate outcrosses to unrelated WT fish, which further minimizes any potential systematic off-target effects. All females used in this study were gravid (egg bearing) based on visual evidence (distended abdomen) and confirmed upon tissue collection. Gravid females were used to ensure physiological reproductive state was consistent across genotypes.

#### 2.1.2. Housing

Female offspring from AR^Het^ × AR^Het^ matings were housed in a mixed-genotype 121 l tank with one size matched WT stimulus male during a three- to six-week period. The WT stimulus male was separated from the females using a clear perforated barrier to prevent mating during the period of stable social interaction, while still allowing the male to perform a full suite of typical social behaviours towards females. This ensured that the females all had the same reproductive status (i.e. they were gravid, displaying distended bellies filled with eggs, easily identifiable and ready to spawn). Following the three- to six-week stable social interaction period, females were chosen at random, euthanized using rapid cervical transection and their brains and blood were collected. A fin clip was collected during tissue collection for use in polymerase chain reaction (PCR)-based genotyping. Due to mating from the stimulus male getting over the border, three female fish had to be excluded from analysis, final *n* = 12.

### 2.2. Tissue collection

Upon removal from their mixed-genotype community tank, females from the ARα^Het^ cross were euthanized in an ice bath slurry before cervical transection, then their brains and blood were collected. We measured standard length (SL) in cm from the tip of the mouth to the base of the tail fin. Body mass (BM) in grams was measured and recorded, and gonads were extracted and weighed. Blood for each animal was collected from the dorsal artery using 2–3 heparin-coated capillary tubes (VWR^®^).

Brains for fish from the ARα^Het^ cross were dissected and fixed in 4% paraformaldehyde (PFA) with a pH of 7.0 in a 20 ml vial for 24 h before being cryoprotected using a sucrose gradient starting with 15% sucrose (in 1× PBS; Gibco) overnight and subsequently 30% sucrose (in 1× PBS; Gibco) for 1–2 days (or until sunk to the bottom of the vial, indicating absorption of sucrose). Once all brains had sunk, they were embedded in Neg-50 (Epredia) and stored at −80°C until sectioning at 30 μm on a cryostat (Thermo Scientific, HM525NX).

### 2.3. Histology

#### 2.3.1. *In situ* hybridization

*In situ* hybridization (ISH) of ARα mutant brains was performed to analyse the expression of the brain aromatase (*cyp19a1*) as previously described [19]. Briefly, PCR was used to amplify a sequence from *cyp19a1* using primers: *cyp19a1* forward, 5′-AGATGATAATCGCAGCCCCC-3′; *cyp19a1* reverse, 5′-TAATACGACTCACTATAGGGGAGTGACCAGGATGGCCTT-3′. The PCR product was subsequently confirmed using Sanger sequencing and transcribed *in vitro* using T7 RNA polymerase (Promega) and Digoxygenin (DIG) labelled dNTPs (Roche), the resultant RNA *cyp19a1* probe was then used for ISH.

Tissue for ISH was cryosectioned coronally at 30 µm (Thermo Scientific, HM525NX) at −20°C and mounted on Superfrost® slides (VWR) and allowed to dry for 2 h before storage at −80°C. All steps of the ISH protocol were performed in a 40 ml Coplin jar unless otherwise noted. Twenty-four hours before first day of ISH, slides were taken out of −80°C storage and allowed to dry at room temperature. ISH for *cyp19a1* was performed using an established protocol similar to one previously described in Lopez & Alward [12] with a few modifications. Briefly, ISH began by fixing the tissue in 4% PFA before serially dehydrating tissue slides with ethanol washes (Thermo Fisher) starting with 50% EtOH, followed by 70% and finally 100%. Next, the slides were allowed to air dry briefly (5 min) to ensure dehydration was complete and to ensure tissue adhesion. ISH proceeded with addition of Proteinase K (10 mg ml^−1^; Life Technologies). Sections were briefly rinsed with 1× PBS pH7.4 (Gibco) and then rinsed again in TEA buffer (0.1 M triethanolamine-HCl; 1 : 400 acetic acid). Slides were then incubated in prehybridization buffer (50% formamide, 5× SSC, 0.1% Tween-20, 0.1% CHAPS and 5 mM EDTA) for 2 h at 62°C and subsequently incubated overnight at the same temperature in hybridization buffer (50% formamide, 5× SSC, 0.1% Tween-20, 0.1% CHAPS, 5 mM EDTA, 1 mg ml^−1^ torula yeast RNA, 100 μg ml^−1^ Heparin and 1× Denhardt’s solution) with 32 ng of DIG-labelled RNA probe. On the next day, slides were washed with 50% formamide (Thermo Fisher) and 2× SSC (Gibco) at 62°C. This was followed by three washes with 2× SSC at 37°C and treatment with RNaseA (200 ng ml^−1^; Qiagen) in 2× SSC at 37°C. The slides were then washed in maleic acid buffer (MABT; 100 mM maleic acid, 150 mM NaCl, 0.1% Tween-20), and subsequently blocked with MABT plus 2% BSA for 1.5 h at room temperature. Anti-DIG antibody fragments conjugated with alkaline phosphatase (Roche; 1 : 5000) were diluted in MABT : 2% BSA and then incubated at 4°C overnight. On day 3, slides were washed with MABT and subsequently with alkaline phosphatase buffer (100 mM Tris pH9.5, 50 mM MgCl_2_, 100 mM NaCl, 0.1% Tween-20, 5 mM Levamisole (Tetramisole)) containing NBT (37.5 mg/ml; Roche) and BCIP (94 mg ml^−1^; Roche) for 3.5 h in a 37°C oven. Slides were then washed in 1× PBS, and cover slipped with Aquapolymount aqueous mounting media.

### 2.4. Microscopy and image analysis

All images of stained *cyp19a1* ISH brains were obtained using Nikon eclipse Ti2 microscope and a DS-Qi2, Fi3 camera set. Images were taken at ×20 magnification using the stitching function to capture the entire coronal section of interest to better quantify *cyp19a1* signal intensity. Consistent exposure settings were used for taking pictures across each of the individual brains as well as across different regions of the brain.

#### 2.4.1. Quantification of *cyp19a1* expression

**Figure 1.**
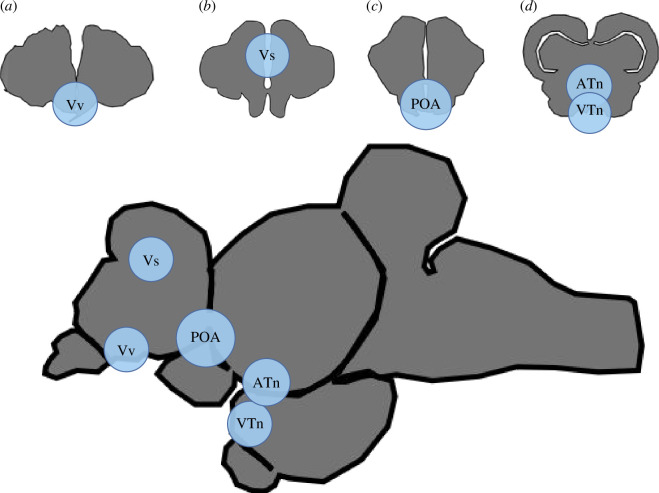
SBN regions for analysis. *Astatotilapia burtoni* coronal brain regions analysed for *in situ* hybridization. (*a*) shows the Vv (homologue of the LS), (*b*) shows the VS (partial homologue of the BNST), (*c*) depicts the preoptic area (POA) and (*d*) shows the ATn (VMH homologue) and the VTn (AH homologue).

We quantified *cyp19a1* expression in various areas of the SBN. In the forebrain, we quantified expression in the following putative homologues of the SBN: the POA, Vs and Vv; and in the midbrain: the VTn and the ATn. Figure 1 shows the different brain regions analysed and their location along the brain.

The final sample sizes for analysis of *cyp19a1* expression across brain regions are as follows: *n* = 12, AR^WT^ = 4, AR^HET^ = 4, AR^KO^ = 4. Thus, for each brain region analysed (Vv, Vs, VTn and ATn) four individuals were analysed per genotype. Analysis of brain regions was done with the free source programme ImageJ (imagej.nih.gov/ij/; RRID: SCR_003070), which allowed for tabulation of expression in regions of interest throughout the brain. The images were first converted to 8-bit black and white images, and a region of interest (ROI) was drawn surrounding the specific brain region of interest. The location of the ROIs for each of the regions analysed was approximated using a previously published brain atlas from Maruska *et al*. [7]. Thus, using this atlas as reference, and the full image of the ISH-treated brains, the landmarks for brain regions of interest were approximated and ROIs were drawn for the specific areas of interest. Once an ROI was selected, the background of the 8-bit images was removed by changing the image threshold using the auto-threshold function on ImageJ and consistently choosing the same threshold settings for mutant or WT brains. Subsequently, the resulting image was subtracted from the original 8-bit picture resulting in a .tiff image file with prominent staining and low/non-present background (hereby called the optimal image). The ROI was once again opened, and the threshold of the optimal image was once again adjusted using the threshold function on ImageJ. The thresholded particles within the ROI of the optimal image were then analysed, and numerical values were obtained detailing the intensity and area of each of the particles within the ROI using the analyse particles function on ImageJ. These values were then added for each hemisphere of the ROI giving the resulting numerical values for each brain region.

### 2.5. Statistics

Statistical analyses were performed using PRISM software (GraphPad Prism version 9.0) to compare *cyp19a1* expression in mutant and WT brains. Specifically, ordinary one-way ANOVAs with Tukey’s multiple comparisons test per group were used to compare differences in WT versus HET, WT versus KO and HET versus KO groups. If equality of variance or normality assumptions were not met, log transforms were used to meet those assumptions. Differences were considered significant at *p* ≤ 0.05.

## 3. Results

### 3.1. ARα deficiency leads to an increase in *cyp19a1* expression in the female *A. burtoni* POA and a decrease in BNST/medial amygdala

**Figure 2.**
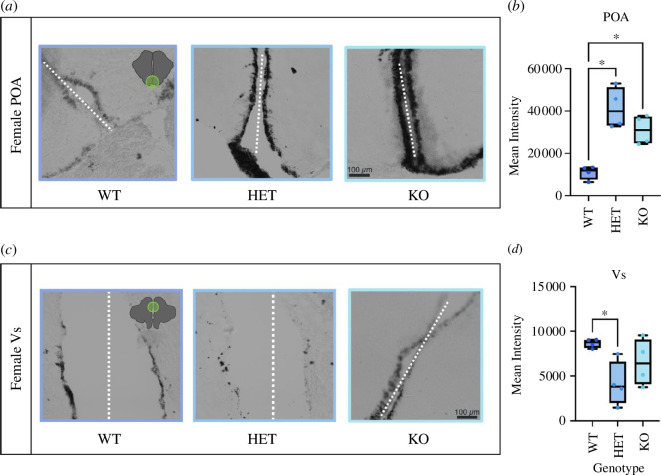
cyp19a1 expression in the female POA and Vs. Panel (*a*) shows representative greyscale images of *in situ* treated brains with *cyp19a1* (brain aromatase) expression in the POA for each of the mutant genotypes as well as the WT. Panel (*b*) depicts *cyp19a1* expression, graphed as mean intensity, for the POA. Note that ARα HET and ARα KO brains have higher *cyp19a1* expression compared with WT; however, the HET and KO genotypes do not differ from each other. Panel (*c*) depicts representative images of the Vs for WT versus ARα HET and ARα KO brains. Panel (*d*) shows *cyp19a1* expression in WT versus mutant genotypes, only the HET genotype showed a significant reduction in *cyp19a1* expression compared with WT. The white dotted line represents the brain midline for all images. Bars on box plot represent max and min points.

We observed a robust effect of genotype on *cyp19a1* expression in the POA (figure 2*a*,*b*). Specifically, ARα^HET^ and ARα^KO^ females had approximately double the intensity of *cyp19a1* expression in the POA compared with ARα^WT^ females (figure 2*b*; *F*_2,9_ = 19.00, *p* = 0.0023, *p*_WT vs HET_ = 0.0100, *p*_WT vs KO_ = 0.0157 and *p*_HET vs KO_ = 0.3247). There was also a significant effect of genotype on *cyp19a1* expression in the Vs, wherein ARα^HET^ females had lower *cyp19a1* expression compared with ARα^WT^ females (figure 2*d*; *F*_2,9_ = 4.659, *p* = 0.0409, *p*_WT vs HET_ = 0.0334, *p*_WT vs KO_ = 0.3668 and *p*_HET vs KO_ = 0.2867). Other brain regions analysed including the Vv, ATn and VTn showed no significant differences between the WT and mutant groups (electronic supplementary material, figure S1; Vv: *F*_2,9_ = 1.84, *p* = 0.2126; ATn: *F*_2,9_ = 1.773, *p* = 0.2243; VTn: *F*_2,9_ = 2.200, *p* = 0.1668).

### 3.2. ARα regulates *cyp19a1* expression in brain regions important for reproductive and aggressive behaviours in female *A. burtoni*

The area of detectable expression for *cyp19a1* was also measured for each of the WT versus mutant genotypes. For this measure, no significant results were found for any of the brain regions (electronic supplementary material, figure S1). Vv: *F*_2,9_ = 0.750, *p* = 0.5195; ATn: *F*_2,9_ = 1.773, *p* = 0.2243; NLT: *F*_2,9_ = 2.200, *p* = 0.1668; electronic supplementary material, figure S1). Vs: *F*_2,9_ = 1.267, *p* = 0.3276; and POA: *F*_2,9_ = 0.4042, *p* = 0.6790 similarly showed no significant differences between the WT and mutant genotypes.

## 4. Discussion

We identified the POA and Vs (partial homologue of the mammalian BNST) as regions where ARα deficiency impacts *cyp19a1* expression in female *A. burtoni*. These results contrast sharply with those in our previous study showing ARα-deficient males possessed lower *cyp19a1* expression compared with WTs only in the ATn [12]. Notably, these brain regions are all involved in different aspects of social behaviour, highlighting a potential mechanism involving ARs, *cyp19a1* and sex steroid hormones like androgens and oestrogens in the production of sex-specific behaviours. The findings here were made in gravid females with different levels of ARα deficiency; it remains to be seen whether reproductive state influences the impact of ARα on *cyp19a1* expression. Below we discuss the implications of our results in the context of potential mechanisms of sexual differentiation of brain and behaviour in *A. burtoni* and other teleosts.

Our results indicate ARα modulates *cyp19a1* expression in the female *A. burtoni* brain. These findings clearly differ with recent results showing the impacts of ARα mutation on *cyp19a1* expression in the male *A. burtoni* brain. Several studies using steroid immersion techniques have identified androgens and oestrogens as critical drivers of sexual differentiation in teleost fishes. Immersing larval teleosts in androgens or oestrogens can lead to anywhere from approximately 75% to 100% male and female fish, respectively [20–35]. In addition, pharmacological inhibition of aromatase by immersion also leads to a majority male offspring in teleosts [23,29,32,36–41]. This suggests that changes in the concentration of androgens and oestrogens affect sexual differentiation of the *A. burtoni* brain. Thus, we hypothesize that organizational effects of sex steroids on the brain during development and/or adulthood in the brain influence the role of ARα on *cyp19a1* expression in a brain region and sex-specific manner. This hypothesis can be tested in future studies in which ARα and *cyp19a1* functions are modulated using tissue- and temporally specific genetic or pharmacological manipulation techniques in both sexes.

Moreover, since ARα co-expresses with *cyp19a1* in both WT male and female *A. burtoni* in the POA, Vs and ATn—the three regions that show sex-specific *cyp19a1* expression in response to ARα deficiency—epigenetic sex-specific differences in accessibility of ARα to the *cyp19a1* promoter may underlie these differences. In rodents, epigenetic processes are intimately tied to sexual differentiation of the brain and behaviour [42,43]. In adult rats, sex differences exist in the ability of the same amount of testosterone to influence aromatase expression in the POA, BNST and VMH [44], all homologues to the brain regions mentioned above. Our results further show that the area of detectable expression does not change for any of the mutant genotypes in either the POA or the Vs of these females, suggesting that there is an increase of *cyp19a1* being produced in these areas for certain genotypes but not a change in the *number* of cells that express *cyp19a1,* further highlighting those regulatory mechanisms mediated by ARα may be disrupted in these mutants. Future studies assessing epigenetic modifications of aromatase in the *A. burtoni* SBN will be important in disentangling these mechanisms.

Interestingly, haplo-deficiency (ARα^HET^) of ARα in the BNST in *A. burtoni* females led to a decrease of *cyp19a1,* while complete deficiency (ARα^KO^) of ARα led to statistically indistinguishable levels of *cyp19a1* expression to WT females. In contrast, the results in the POA for these same females showed elevated levels of *cyp19a1* for both ARα^HET^ and ARα^KO^ in comparison with WT females. This suggests the possibility that the mechanisms regulating *cyp19a1* expression in different brain regions may differ based on potential compensatory processes that occur depending on whether and to what degree ARα is present. One potential mechanism could involve the second AR paralog, ARβ, compensating for ARα. Future studies should investigate whether and how either AR paralog may be modified depending on the presence of the other.

The POA and BNST are involved in the generation of sex-specific social behaviours across vertebrate species [5,45–59], providing potential clues into what traits ARα modification of *cyp19a1* expression in these areas could affect. Based on recent work, ARα is not involved in the generation of female aggression [60]. However, previous findings have shown oestrogenic signalling in the BNST and POA is involved in female-typical mating, suggesting that ARα-induced alteration of *cyp19a1* expression in the POA may impact mating behaviours. For example, a recent study in mice found that the BNST shows sex-specific transcriptional programmes governed by oestrogenic signalling [61]. In medaka, sexually dimorphic gene expression is present in the POA and Vs [53,62] including for the oestrogen receptor gene *esr2b*, which is expressed at higher levels in females than males and nearly exclusively in the POA and Vs [62]. Female medaka lacking functional *esr2b* do not perform female-typical mating behaviours and produce male-typical mating behaviours instead [62], suggesting that oestrogenic signalling within the POA and Vs is critical for the production of female mating behaviours in teleosts. Thus, it is possible that changes in *cyp19a1* in the Vs and POA modified by ARα could lead to changes in the levels and utilization of estradiol and its receptors leading to important changes in mating behaviour. Future studies using *cyp19a1*-deficient fish will help elucidate the specific roles oestrogens and aromatase play in the production of sex-typical social behaviours, and how changes in *cyp19a1* may change sex-typical brain sexual differentiation. The current findings provide a potential molecular and neural roadmap involving androgens, ARα, brain aromatase and oestrogenic signalling that may shape female typical brain and behaviour. Dissecting these mechanisms may lead to novel insights on the control of behaviour by steroid hormone-modulated genes.

### 4.1. Limitations

We used *in situ* hybridization staining as a way to measure and compare expression of the *cyp19a1* between ARα mutant genotypes (WT versus HET or KO). Other methods of quantifying gene expression and further studies should be done using quantitative methods like qPCR and/or RNA sequencing of specific brain regions in ARα mutants [63]. Such methods would allow for cross-methodological replication of the effects of ARα mutation on *cyp19a1* expression.

## Data Availability

Data and relevant code for this research work are stored in GitHub [64] and have been archived within the Zenodo repository [65]. The supplementary file includes additional background on sample sizes, the area of detectable expression for *cyp19a1* and graphs of results for all SBN brain regions not included in the manuscript [66].
